# An In Vitro Evaluation of the Molecular Mechanisms of Action of Medical Plants from the Lamiaceae Family as Effective Sources of Active Compounds against Human Cancer Cell Lines

**DOI:** 10.3390/cancers12102957

**Published:** 2020-10-13

**Authors:** Przemysław Sitarek, Anna Merecz-Sadowska, Tomasz Śliwiński, Radosław Zajdel, Tomasz Kowalczyk

**Affiliations:** 1 Department of Biology and Pharmaceutical Botany, Medical University of Lodz, 90-151 Lodz, Poland; 2 Department of Economic Informatics, University of Lodz, 90-214 Lodz, Poland; anna.merecz-sadowska@uni.lodz.pl (A.M.-S.); radoslaw.zajdel@uni.lodz.pl (R.Z.); 3 Laboratory of Medical Genetics, Faculty of Biology and Environmental Protection, University of Lodz, 90-236 Lodz, Poland; tomasz.sliwinski@biol.uni.lodz.pl; 4 Department of Molecular Biotechnology and Genetics, University of Lodz, 90-237 Lodz, Poland; tomasz.kowalczyk@biol.uni.lodz.pl

**Keywords:** Lamiaceae, cost-effective natural products, secondary metabolites, cancer, molecular mechanisms of action, in vitro studies

## Abstract

**Simple Summary:**

Plants have been used in folk medicine for thousands of years. The Lamiaceae family is one of the largest families of flowering plants and includes a wide variety of species with biological and medical uses. They are mainly herbs and shrubs with an aromatic scent and rich in valuable compounds of great value in medicine. The article focuses on the assessment of the anticancer properties of extracts, essential oils, and pure compounds derived from various species of the Lamiaceae family and their potential molecular mechanisms of action in in vitro studies against the four most common types of cancer in women and men: breast, lung, prostate, and colon.

**Abstract:**

It is predicted that 1.8 million new cancer cases will be diagnosed worldwide in 2020; of these, the incidence of lung, colon, breast, and prostate cancers will be 22%, 9%, 7%, and 5%, respectively according to the National Cancer Institute. As the global medical cost of cancer in 2020 will exceed about $150 billion, new approaches and novel alternative chemoprevention molecules are needed. Research indicates that the plants of the Lamiaceae family may offer such potential. The present study reviews selected species from the Lamiaceae and their active compounds that may have the potential to inhibit the growth of lung, breast, prostate, and colon cancer cells; it examines the effects of whole extracts, individual compounds, and essential oils, and it discusses their underlying molecular mechanisms of action. The studied members of the Lamiaceae are sources of crucial phytochemicals that may be important modulators of cancer-related molecular targets and can be used as effective factors to support anti-tumor treatment.

## 1. Introduction

Cancer is an important health problem and leading cause of death globally. The development of cancer is a multistage process that begins with genetic alteration and is followed by abnormal cell proliferation. Carcinogenesis is strictly related to the activation of oncogenes (induction of cell growth) and the inactivation of tumor suppressor genes (repression of cell growth), resulting in a loss of control of cell cycle progression. This initiation stage is followed by progression related to additive mutation within cells, some of which are implicated in even more rapid growth, and the suppression of cancer cell death. As a result of these changes, mature epithelial cancer cells may undergo epithelial–mesenchymal transition (EMT), which is characterized by the reduction of adhesion among cells and increased cell motility. Finally, tumor invasion and metastasis occur, and these are strictly related to angiogenesis, i.e., the process of new blood vessel formation. The progression of angiogenesis depends on the secretion of growth factors by cancer cells. The transformation of normal cells into malignant ones is a complex process regulated at every step by numerous factors, each of which may be a crucial target for anticancer agents [1,2].

The most prevalent form of cancer globally is lung cancer (11.6% of total cases), followed by breast cancer, among women (11.6%), prostate cancer, among men (7.1%), and colorectal cancer (6.1%) [3]. In 2018, more than 18 million cases of cancer were reported, and there were more than 9.6 million deaths globally [4]. In addition, the cost of medical care and economic value of nonmedical expense has been still rising [5]. 

Research into cancer therapy is currently directed toward the development of new therapeutic strategies and more effective chemotherapeutic factors [6]. An important source of novel effective agents with medicinal potential is the plant kingdom [7]. It is estimated that approximately 35 to 70,000 higher plant species produce secondary metabolites with diverse forms of bioactivity that are widely used in the regulation of signaling and metabolic pathways [8]. One family of particular interest is the Lamiaceae, which includes a number of herb and shrub genera with significant anticancer activities, such as *Salvia* sp., *Thymus* sp., *Origanum* sp., *Melissa* sp., *Plectranthus* sp., or *Scutellaria* sp.; all have been found to possess effective antiproliferative potential against lung, breast, prostate, and colon cancer cells in vitro. They commonly exert their cytotoxicity by promoting cancer cell death, especially via the apoptosis pathway, but they have also been found to influence angiogenesis [9]. Therefore, plant extracts, individual compounds, and essential oils from the Lamiaceae may support treatment as alternative or complementary cancer therapy.

The present paper focuses on the anticancer effects of plant extracts, purified single compounds, and essential oils from selected species of the Lamiaceae family. It discusses their in vitro cytotoxicity toward lung, colon, breast, and prostate cancer cell lines and the underlying mechanisms of action. 

## 2. Criteria for Selection of Experimental Papers

This review was conducted to report work done previously to access the anticancer activity of plants from the Lamiaceae family published from 2015 to 2020. The studies were selected in the electronic databases PubMed/MEDLINE, Scopus, Web of Science, and Google Scholar. The search terms included Lamiaceae alone, and with the following: plant extract, derived compounds, essential oils, cancer, lung cancer cells, colon cancer cells, breast cancer cells, prostate cancer cells, mechanism of action. Published experimental studies reporting extracts, derived compounds, and essential oils from plants belonged to the Lamiaceae family with in vitro activity against lung, colon, breast, and prostate cancer cell lines were included. Research reporting review articles, published in languages other than English, abstract only or without full text access, lacking specific plant names with no reports of clear objective and methodologies, published more than five years ago, using plant species other than Lamiaceae, and cell lines other than lung, colon, breast, and prostate were excluded. The duplicates of articles obtained from the electronic databases were removed. After removal, inclusion/exclusion criteria were checked. Each selected document was examined and the following data were extracted and presented in the table: the scientific names of the species, parts of the plants used for extract preparation, types of extract, class of compounds, or compounds identified in extracts, cancer cell line, and reference. Articles with included mechanisms of action of interested plant extracts, single compounds, and essential oils were discussed in the main text.

## 3. Cancer

The term cancer is used to refer to a large group of diseases that can affect any part of the body. They are caused by uncontrolled cell proliferation that can take place in different tissues and spread into surrounding and distant organs [10,11]. Cancer occurs by a series of successive mutations in the relevant genes, leading to changes in cell function. Various physical and chemical factors play an obvious role in the formation of gene mutations and the appearance of cancer cells [12]. 

The first records of cancer date back to the ancient Egyptian and Greek civilizations, where the disease was treated mainly with radical surgery and often ineffective cauterization procedures, leading to the death of patients [13]. Currently, cancer is one of the most commonly occurring conditions and a major public health problem worldwide [14,15,16]. In 2018, cancer was responsible for approximately 9.6 million deaths [4]. Statistics show that high cancer morbidity and mortality are associated with an increasing incidence of risk factors such as overweight, alcohol abuse, smoking, unhealthy diets, urban air pollution, hepatitis and human papilloma virus, lack of physical activity, or sedentary lifestyle [17,18]. The World Health Organization (WHO) reports that in 2018, the highest percentages of cancer types in men occur in the lung, prostate, and colon, while the greatest prevalence in women is observed in the lung, breast, and colon. The most common types in both men and women were lung (2.09 million cases) and colon cancer (1.80 million cases). The second most common types of cancer were breast cancer in women (2.09 million cases) and prostate cancer in men (1.28 million cases) [14,19,20]. 

There are two main forms of lung cancer: non-small cell lung cancer (NSCLC; 85% of patients) and small cell lung cancer (SCLS, 15%). In 2018, this cancer caused 1.76 million deaths [14]. It is currently the most lethal malignant tumor in the world, often because it is not detected until the disease progresses significantly, leading to a significant reduction in the patient’s quality of life [21,22]. So far, genetic factors leading to increased susceptibility to lung cancer have been poorly studied. First-degree relatives of patients with lung cancer are at increased risk, even after adjusting for smoking habits. The most commonly accepted causes of lung cancer include active cigarette smoking, exposure to passive smoking, pipe and cigar smoking, indoor and outdoor air pollution, radiation, and occupational exposure to factors such as asbestos, nickel, or chromium [23,24,25]. Among others, important common driver gene mutations in patients with NSCLC are as follows: tumor suppressor genes *TP53* and *PTEN*; *EGFR,* which encodes protein, is involved in cell growth and survival [26]. These genes constitute molecular targets for compounds from the Lamiaceae family. 

Another common cancer among women, and one of the leading causes of death, is breast cancer [27]. In 2018, this cancer caused 627,000 deaths [14]. Breast cancer risk is related to many factors including a diet too high in calories leading to obesity, tobacco smoking, hormonal history, exposure to estrogen and progesterone, early first menstruation, and late menopause. All of these factors lead to shorter menstrual cycles and hormone exposure [28]. Genetic predisposition also plays an important role, as approximately 7% of breast cancer cases are hereditary [29]. In turn, mutations in the *BRCA1* and *BRCA2* genes cause as much as 80% of genetic breast cancer [30,31]. BRCAs proteins regulate genes linked to DNA repair, the cell cycle, and apoptosis [32]. Another crucial mutations is that in *TP53*, which is linked with histological grade in breast cancer patients [33]. 

Prostate cancer is the second most common malignant neoplasm after lung cancer in men around the world [34]. In 2018, this cancer caused 358,989 deaths, this being 3.8% of all deaths caused by cancer in men. About 60% of cases are diagnosed in men over 65. The mean age of diagnosis is 66 years. The disease rarely occurs in people under 40 years of age [35]. The main risk factors for the development of prostate cancer in men can be divided into non-modified and modified factors. Non-modified factors include age, family history, ethnicity, and genetic factors, while the modified ones are obesity, infectious diseases, smoking, hormones, or occupational and external exposure [36,37,38]. Inherited mutations in specific genes, such as *BRCA1*, *BRCA2*, and *HOXB13*, are the cause of some hereditary prostate cancer cases. HOXB13 protein regulates androgen receptor function [39]. Men with mutations in these genes are at high risk of developing prostate cancer and, in some cases, other cancers during their lifetime [40]. Additionally, other different genes have been implicated in prostate cancer by genetic alteration, depending on types (familial, sporadic, hereditary): *TP53, PTEN,* and protooncogenes *MYC,* which also constitute molecular targets for compounds from the Lamiaceae family [41].

Colorectal cancer is already the second most common cause of cancer death in the world (862,000 deaths) and 65% of all new incidences occur in high-income countries in Europe [14]. This incidence constitutes a 1.51% cumulative risk (assuming the absence of other competing causes of death) of colon cancer among men age 0–74 years, and a 1.12% risk among women. Their cumulative lifetime risks are 1.2% and 0.65%, respectively [3]. Colorectal cancer has been attributed to a number of mutations: mutational inactivation of tumor suppressor genes such as *TP53*, and activation of the oncogene pathway *PI3K*. These genes constitute molecular targets for compounds from the Lamiaceae family [42]. Genetically, colorectal cancer is divided into three categories: sporadic (about 60%) for patients with no family history, family (about 30%) for patients with at least one relative with colorectal cancer or adenoma, and hereditary forms (about 10%) due to the germline inheritance of mutations [43,44]. The development of colorectal cancer is believed to be supported by family history, lack of physical activity, old age, excessive alcohol consumption, high-fat diet, diabetes, and inflammatory bowel diseases, including ulcerative colitis and Crohn’s disease [45,46,47].

## 4. Cancer and Plants

Plants have been used in folk medicine for thousands of years [48]. The oldest written evidence of the use of medicinal plants in the preparation of medicines was found on a Sumerian Nagpur clay plate, approximately 5000 years old. It contained 12 prescriptions for the preparation of drugs, relating to over 250 different plants, including genera such as *Papaver* sp., *Hyoscyamus* sp., or *Mandragora* sp. containing alkaloids [49]. Medicinal plants are still used in developing countries as the source of healing due to their natural multidirectional action and low cost. The secondary metabolites contained in plants mainly do not play a direct role in their growth but serve as defence compounds against herbivores and microorganisms; therefore, they are required for survival in a specific environment. Most are polyphenols, alkaloids, terpenoids, or polyketides, among which there are a number of subclasses. Some have been found to demonstrate anticancer [50], anti-inflammatory [51], antioxidant [52], antimicrobial [53], antiviral [54] or anti-obesity effects [55]. In addition, while they can act as pure compounds, they also can show synergistic or antagonist effect with enhanced, or weaker, biological properties when administered as plant extracts [56,57,58]. 

According to the WHO, some nations still consider plant-based treatments to be the primary source of medicine and developing countries are exploiting the benefits of natural origin compounds for therapeutic purposes [59]. Numerous studies confirm the positive use of such natural compounds in the treatment of many diseases [60]. In turn, medicinal plants are also a valuable source of bioactive anticancer agents [61,62,63,64,65,66]. If plant-derived compounds can demonstrate selectivity in research, are non-toxic to normal cells, and show cytotoxicity in cancer cell lines, these compounds can be used in clinical trials for further therapeutic development. However, it is worth remembering that chemicals of plant origin may also exert toxic effects on both animals and human organisms in in vivo study [61,67].

The modern search for anticancer drugs from plants began in the 1950s, with the discovery of vinca alkaloids from the genus *Vinca* [68]. This highlights that medicinal plants remain a source of new drugs [69], e.g., vinblastine or vincristine obtained from the *Vinca* sp., paclitaxel from the *Taxus* sp., camptothecin from the *Camptotheca* sp., or podophyllotoxin from *Podophyllum* sp. [48,61,70,71]. These compounds often exert their anticancer activity by inhibiting the proliferation of cancer cells and inducing cell death [67]. Many in vivo and in vitro studies show the activation of apoptosis in various cancer cells, both under the influence of pure compounds and plant extracts [71]. Numerous plant species from different families (Lamiaceae, Fabaceae, Asteraceae, Papaveraceae, Apocynacea etc.) are traditionally used to treat or prevent the development of cancer.

## 5. The Lamiaceae Family Plants

**Figure 1:**
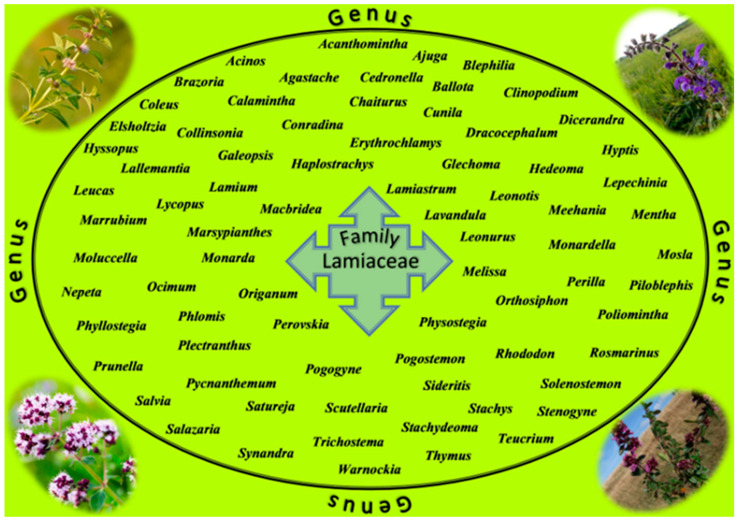
Selected genera belonging to the Lamiaceae family.

The Lamiaceae family is one of the largest families of flowering plants and includes a wide variety of species with biological and medical uses. They are mainly herbs and shrubs with an aromatic scent and rich in valuable compounds of great value in natural medicine. Plants of this family are characterized by square stems and opposite leaves [72,73]. The most famous representatives are thyme, mint, oregano, basil, sage, savory, rosemary, hyssop, and lemon balm, which are used as aromatic spices, and some others with more limited uses [74]. Historically, species in the Lamiaceae family have enjoyed a long history of use in flavoring, preserving food, and for medicinal purposes. This family includes about 250 genera and about 7000 species, with the largest genera being *Salvia*, *Scutellaria*, *Stachys*, *Plectranthus*, *Hyptis*, *Teucrium*, *Vitex*, *Thymus*, or *Nepeta* (Figure 1). It is one of the most economically important families with great diversity and cosmopolitan distribution due to the aromatic properties of most of its members [75]. It is well known that each species produces a wide variety of secondary metabolites with strong antibacterial, antioxidant, anti-inflammatory, antiviral or anticancer properties; the oils comprise a complex mixture of bioactive compounds, in which each ingredient contributes to its overall bioactivity [56]. 

### 5.1. The Lamiaceae Family as a Source of Valuable Secondary Metabolites with Anti-Cancer Potential

The first and largest group of secondary metabolites occurring in the Lamiaceae is polyphenols, which are characterized by at least one aromatic ring having hydroxyl groups. Based on their numbers of phenolic groups and structural elements, polyphenols can be divided into phenolic acids, flavonoids, stilbenes, lignans, lignins, coumarins, anthraquinones, and xanthones [76,77,78]. In the human body, polyphenols exhibit antioxidant properties, which protect against chronic diseases caused by free radicals damaging of tissues and organs [72], as well as various anticancer [79] anti-diabetic [80], neuroprotective [81], anti-inflammatory [82], antiviral [83], antifungal [84] or antibacterial properties [85]. Polyphenols are believed to cause cancer cell death by apoptosis through several mechanisms, such as DNA fragmentation, alteration of the level of apoptotic proteins, and mitochondrial membrane potential and cell cycle arrest [86,87]. 

Another potent group of secondary compounds is the terpenes, in particular oxygenated terpenoids [88]. In turn, terpenoids are categorized into monoterpenes, sesquiterpenes, diterpenes, sesterpenes, and triterpenes depending on the number of their isoprene units. Diterpenes constitute a diverse class of plant metabolites with more than 10,000 different structures, isolated in the Lamiaceae present about 50 different skeletons [89,90]. Most diterpenes play a critical role in ecological interactions of plants and show interesting biological activities both in vitro and in vivo [91,92], such as antibacterial, antifungal, antiprotozoal, enzyme-inducing, anti-inflammatory, and the modulation of immune cell function and anticancer properties [92,93]. Studies have shown that some diterpenes have significant cytotoxic and cytostatic effects on various cell lines of human origin, interfere with the biochemical pathways of apoptosis and the cell cycle phase, and can influence the expression of proto-oncogenes such as avian myelocytomatosis viral oncogene homolog (c-Myc) and B-cell lymphoma 2 (Bcl-2) [93,94]. 

Many members of the Lamiaceae also produce alkaloids, which is an extremely diverse chemical group based on a ring structure including a nitrogen atom. Many alkaloids are toxic and are used by plants to protect themselves against aggression from other organisms [95,96]. Many such compounds have a strong cytotoxic effect and induce apoptosis through various pathways in different cell lines [97,98].

Essential oils (EO) are volatile and complex mixtures of diverse compounds, typically with a strong odor, synthesized as secondary metabolites by aromatic plants, mainly from the Lamiaceae family (132). Although EO are mainly contained in the leaves, they can be found in all the above-ground parts of plants. They are widely used in cosmetics, flavors, fragrances, perfumes, pesticides, and the pharmaceutical industry [99]. These phytocomplexes can be obtained by hydro or dry distillation. Their ingredients include sesquiterpenes, oxygenated sesquiterpenes, monoterpenes, oxygenated monoterpenes, and phenols, among others [100,101]. Many preclinical studies have found some anticancer, antibacterial, antioxidant, or anti-inflammatory effects in a range of cellular and animal models [102], and they have examined their mechanism of action and pharmacological targets [101]. 

**Figure 2:**
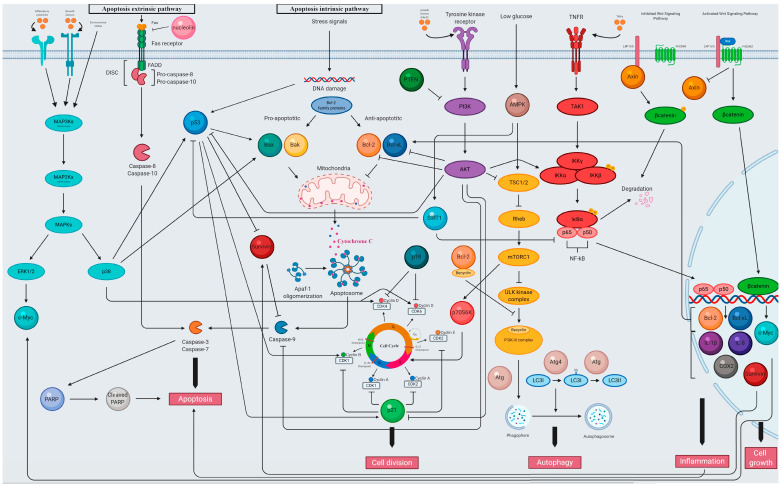
Selected molecular mechanisms of action of extracts, single derived compounds and essential oils from the Lamiaceae family against lung, breast, prostate, and colon cancer cell lines (created by BioRender). Abbreviations: Akt—protein kinase B, AMP—adenosine monophosphate, AMPK—AMP-activated protein kinase, Apaf—apoptotic protease activating factor, Atg—autophagy-related protein, Bak—Bcl-2 homologous antagonist/killer, Bax—Bcl-2 associated X protein, Bcl-2—B-cell lymphoma 2, Bcl-xL—B-cell lymphoma-extra large, CDK—cyclin-dependent kinase, c-Myc—avian myelocytomatosis viral oncogene homolog, COX—cyclooxygenase, DISC—death-inducing signaling complex, ERK—extracellular signal-regulated kinase, FADD—Fas-associated death domain, Her2—human epidermal growth factor receptor 2, IKK—IkB kinase, IL—interleukin, LC3—autophagosomal membrane-associated protein light chain 3, LPR 5/6—low-density lipoprotein receptor–related protein 5/6, MAP2K—MAPK kinase, MAP3K—MAP2K kinase, MAPK—mitogen-activated protein kinase, PARP—poly(ADP-ribose) polymerase, PI3K—phosphatidylinositol 3-kinase, PI3K-III—class III PI3K complex 1, PTEN—phosphatase and tensin homolog, Ras—rat sarcoma viral oncogene homolog, RHEB—Ras homolog enriched in brain, SIRT1—Sirtuin, TAK—TGF-β-activated kinase, TNF—tumor necrosis factor, TNFR—tumor necrosis factor receptor, TSC 1/2—tuberous sclerosis proteins 1 and 2, ULK—Unc-51 like autophagy activating kinase, Wnt—wingless-related integration site. Arrows: Activation— 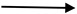; Inhibition— 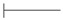.

The particular signaling pathways and related factors that constitute a target for natural anticancer modulators from Lamiaceae discussed in this paper are given in Figure 2.

### 5.2. The Anti-Cancer Activity of Plant Extracts from the Lamiaceae Family

Many members of the Lamiaceae have demonstrated considerable efficacy in inhibiting cancer cell growth through synergistic effects. Extracts from many species demonstrate cytotoxic properties against lung, colon, breast, and prostate cancer cells (Table 1). This section discusses their mechanisms of action, which are mainly based on inducing the apoptosis in cancer cells including lung, breast, prostate, and colon cancer.

#### 5.2.1. The Activity of Plant Extracts from the Lamiaceae Family as Modulators of Cell Cycle

The cell cycle events controlling cell duplication and arrest are highly dysregulated in cancer cells. Cell cycle arrest is closely related to the G1/S, G2/M and M phases’ checkpoints perturbations. Progression is mediated by the activation and deactivation of cyclin-dependent kinases (CDKs), while activation depends on the presence of activated subunits named cyclins. The levels of cyclins and CDKs are changed in human cancers; for example, the levels of CDK1 and cyclin B1 are reduced in human breast adenocarcinoma (MCF-7) and human colorectal cancer (HCT-116) cell lines following treatment with *Micromeria fruticosa* aerial part extract, resulting in G2/M arrest [143,144]. In addition, treatment with *Melissa officinalis* leaf extract blocked the expression of CDKs 2, 4, and 6 and cyclin D3 in the human colon carcinoma (HT-29 and T84) cell lines, and specifically activated an important CDK inhibitor named p18 [145]; in addition, treatment with *Vitex rotundifolia* fruit extract downregulated cyclin D1 and CDK 4 levels in HCT-116 and human colon adenocarcinoma (SW480) [146]. Manipulation of the cell cycle may induce an apoptotic response [147]. *Ocimum basilicum* leaf extract induced cell cycle arrest in MCF-7 cells [148], and *Salvia miltiorrhiza* root extract induced a G2/M phase arrest in human lung adenocarcinoma (Glc-82) cells [149], as did *Melissa officinalis* leaf extract in HT-29 and T84 cells and *Prunella vulgaris* root extract in human breast carcinoma MCF-5 cells [150]. *Satureja khuzistanica* extract enriched with rosmarinic acid [151] was found to increase the size of the apoptotic sub-G0/G1 population in MCF-7 cells.

#### 5.2.2. The Activity of Plant Extracts from the Lamiaceae Family as Modulators of Apoptosis Signaling

Apoptosis or programmed cell death is a crucial mechanism for maintaining cell homeostasis. The process can be triggered by various conditions, including immune reaction or reactive oxygen species (ROS). The study of *Ocimum sanctum* roots extract suggested that it may increase ROS production in the HCI-H460 lung carcinoma cell line and may decrease viability via apoptosis. The same phenomenon is observed for *Melissa officinalis* leaf extracts against HT-29 and T84 cell lines [145]. That strategy is confirmed by the occurrence of excessive numbers of apoptotic cells [152]. It was concluded that also stress originating from the endoplasmic reticulum may play an important role in triggering apoptosis; for example, *Scutellaria barbata* extract induced human lung cancer (CL1-5) cell death [153].

The signaling mechanisms of apoptotic cell death are divided into two pathways: an intrinsic pathway mediated by mitochondria and an extrinsic one mediated by death receptors. Cell size reduction, membrane blebbing, and apoptotic bodies were observed in HT-29 cells after *Stachys pilifera* leaf extract treatment; these were all signs of apoptosis [154]. MCF-7 cells showed a modified nucleus following incubation with *Caryopteris x clandonensis* stem extract [155], and changes in cell rounding, shrinkage, and detachment from other cells after treatment with *Teucrium mascatense* whole plant extract [156]. Another important future of apoptosis is the translocation of phosphatidylserine phospholipid from the inner to the outer plasma membrane, resulting *inter alia* in recognition by phagocytes [157]; this was observed against HT-29 and T84 cell lines after the administration of *Teucrium mascatense* extract [156] and *Melissa officinalis* leaf extract [145]. *Teucrium flavum* whole plant extracts were found to induce DNA fragmentation in breast carcinoma cells (MDA-MB-231) [158], as well as *Vitex rotundifolia* leaf extract in human breast cancer T-47D cells [159] and *Ocimum sanctum* leaf extract in human prostate adenocarcinoma LNCaP cells [160]; such DNA cleavage is a hallmark of apoptosis. *Perovskia abrotanoides* flower extract has also demonstrated proapoptotic effects against MCF-7 cells [161] and *Salvia chorassanica* root extract against MCF-7 and prostate cancer cells (DU-145) [162].

The treatment of human non-small cell lung carcinoma cells (NCI-H460) with *Ocimum sanctum* root extract [152] and MCF-7 cells with *Teucrium sandrasicum* leaf extract [163] resulted in increased mitochondrial membrane permeability, which is characteristic of the intrinsic pathway. The Bcl-2 protein family also induces a loss of mitochondrial membrane potential. This family is separated into two groups: one, including Bak and Bax, which possesses proapoptotic potential, and another, including Bcl-2 and Bcl-xL, which has antiapoptotic activities. The balance between the two groups influences the progression of apoptosis. The Bcl-2 protein family was found to be modulated by the *Origanum compactum* aerial part extract in A549 cells [164]. Changes in the level of Bcl-2 family members may result in the release of numerous pro-apoptotic molecules. The expression of Bcl-2 protein family members in different lines of human lung cancer cells became unregulated following the administration of extracts from *Salvia milithoriza* roots (Glc-82 cells) [149], *Scutellaria baicalensis* root (H358 and H2087 cells) [165], and *Melissa officinalis* leaves (A549) [166]. A similar result was observed for several human colon cancer cell lines treated with *Coleus amboinicus* leaves (WiDr cells) [167] and *Vitex rotundifolia* fruit (HCT-116, SW480, LoVo and HT-29 cells) extracts [146]. In addition, apoptotic signals have been triggered in breast cancer cell lines after incubation with extracts of *Plectranthus amboinicus* leaves (MCF-7 cells) [120], *Orthosiphon stamineus* leaves (MCF-7 cells) [168], *Melissa officinalis* leaves (MCF-7 cells) [166], *Vitex rotundifolia* leaves (T-47D cells) [159], and *Prunella vulgaris* root (MCF-5 cells) [150]. Changes in the expression level of Bcl-2 family protein are also induced in prostate cancer cell lines by extracts of *Ocimum sanctum* leaves (LNCaP cells) [160], *Dracocephalum palmatum* (PC-3 cells) [169], and *Melissa officinalis* leaves (PC-3) [166].

The insertion of Bax/Bak into the mitochondrial membrane results in the formation of a pore complex and release of cytochrome c into the cytosol from the intermembrane space. In contrast, Bcl-2 and Bcl-xl prevent the release of cytochrome c. Excessive levels of cytochrome c were observed in MCF-7 cells after *Orthosiphon stamineus* leaf extract treatment [168]. Cytochrome c binds to apoptotic protease-activating factor 1 (Apaf-1) to create an apoptosome that activates caspase-9. Caspase-9 was found to be upregulated following treatment with *Coleus amboinicus* leaf extract in WiDr cells [167], *Teucrium sandrasicum* leaf extract in MCF-7 cells [163], *Vitex rotundifolia* leaf extract in T-47D cells [159], *Micromeria fruticose* aerial part extract in MCF-7 cells [144], *Stachys pilifera* leaf extract in HT-29 cell line [154], and *Teucrium chamaedrys* aerial and flowering parts in SW480 cells [170].

Caspase-9 activity results in the activation of the effectors caspase-3 and caspase-7. Caspase-3 and/or caspase-7 activity were observed in the Glc-82, LNCaP, MCF-7, T-47D, MCF-7, and WiDr cell lines following treatment with, respectively, extracts of *Salvia miltiorrhiza* roots [149], *Ocimum sanctum* leaves [160], *Plectranthus amboinicus* leaves [120], *Vitex rotundifolia* leaves [159], *Teucrium mascatense* whole plant [156], and *Coleus amboinicus* leaves [167]. The induction of caspase-3 and/or caspase-7 cleavage was also observed for *Melissa officinalis* leaf extracts against HT-29 and T84 cells [145] and for *Origanum majorana* leaf extract against HT-29 cells [171]. *Dracocephalum palmatum* leaf extract also induced apoptosis according to the intrinsic pathway via the upregulation of activated caspase-3 in PC-3 cells [169]; the same was observed for *Nepeta cataria* aerial parts on PC-3 cells [172] and *Scutellaria baicalensis* whole body extract on H358 and H2087 cell lines [165].

In apoptosis, the detection of poly-ADP-ribose-polymerase (PARP) is an important diagnostic method because it produces specific patterns of proteolytic cleavage fragments [173]. Increased expression of PARP1, a hallmark of apoptotic death, was observed in Glc-82 cells treated with *Salvia miltiorrhiza* roots extract [149], LNCaP cells incubated with *Ocimum sanctum* leaf extract [160], and PC-3 cells interacted with *Nepeta cataria* aerial part extract [172]. PARP activation was also demonstrated after the treatment of *Teucrium mascatense* whole plant extract in MCF-7 cells [156], *Rosmarinus officinalis* leaf extract in A549 cells [174], and *Scutellaria baicalensis* root extract in H358 and H2087 cells [165], suggesting the involvement of mitochondria in the apoptotic signals.

The extrinsic pathway is initiated by the TNF (tumor necrosis factor), TRAIL (TNF-related apoptosis-inducing ligand), and Fas ligands binding to the extracellular domain of death receptors, such as type 1 TNF receptor (TNFR1), TRAIL, and Fas receptors. The *Teucrium chamaedrys* aerial flowering part extract initiated an excessive expression of Fas in SW480 cells [170]. The Fas ligand–receptor junction is inhibited by nucleolin, which is a protein that protects against apoptotic death [175]. *Orthosiphon stamineus* leaf extract treatment significantly decreased the nucleolin level in MCF-7 cells [168]. The attachment of a Fas to a specific receptor triggers the formation of specific death-inducing signaling complex (DISC) possessing a Fas-associated death domain (FADD); this complex can recruit an adaptor protein and then activate initiator caspase-8 and caspase-10. Caspase-8 induction was found to induce apoptosis in MCF-7 and HCT-116 cells following *Micromeria fruticose* aerial part extract administration [144], as well as treatment with *Vitex rotundifolia* leaf extract in T-47D cells [159], *Stachys pilifera* leaf extract in the HT-29 cell line [154], and for *Teucrium chamaedrys* aerial flowering part extract in SW480 cells [170]. In addition, it was speculated that *Satureja khuzistanica* extract enriched in rosmarinic acid can induce apoptosis through activation of the extrinsic pathway in MCF-7 cells [151]. Then, the activation of caspase-8 and caspase-10 is followed by the activation effector caspases 3/7, enzymatic cleavage of numerous downstream targets, and cell death [176,177]. The Fas-mediated apoptosis pathway is believed to play a role in *Scutellaria barbata* extract-induced CL1-5 cell cytotoxicity [153].

An important inhibitor of apoptosis is called survivin. This protein is under-expressed in cancer cells and is related to poor clinical outcome through the blockage of apoptosis by caspase inhibition [178]. Significant reductions of survivin levels were observed for *Micromeria fruticosa* aerial part extract in MCF-7 and HCT-116 cell lines [143,144], *Origanum majorana* leaf extract in HT-29 cells [171], and for *Scutellaria barbata* whole plant extract in HT-29 cells [179]. Another key molecule that plays a crucial role in the negative regulation of apoptosis is Her2, belonging to the human epidermal growth factor receptor family. Excessive Her2 expression is related to antiapoptotic signals via the activation of survivin and Bcl2 protein. *Melissa officinalis* extract shows significant potential to reduce Her2 levels in PC-3 cells [166].

Another regulator of apoptosis and autophagy is mammalian target of rapamycin kinase (mTOR), which also demonstrates pleiotropic features [180]. The activation of the mTOR pathway was promoted by *Ocimum basilicum* leaf extract in MCF-7 cells with p70S6K, which is a downstream target kinase [181]. Similar features were shown for *Scutellaria baicalensis* root extract in H358 and H2087 cell lines [165].

#### 5.2.3. The Activity of Plant Extracts from the Lamiaceae Family as Modulators of p53 Signaling

DNA damage occurs due to metabolic processes and environmental factors including ROS and results in an increase in the levels of tumor suppressor protein p53. The protein serves as a key regulator of the cellular response and triggers target genes including CDK and inhibitor p21, taking part in cell cycle arrest, and the proapoptotic protein Bax [182]. p53 expression was dysregulated in WiDr cells by *Coleus amboinicus* leaf extract [167], and the levels of both p53 and p21 increase in Glc-82 after treatment with *Salvia miltiorrhiza* root extract [149] and in MCF-7 cells after treatment with *Plectranthus amboinicus* leaf extract [120]. The level of p21 was induced in cell lines HT-29 and T84 after incubation with *Melissa officinalis* leaf extract [145]. p53 may be regulated by Sirtuin 1 (SIRT1), which is a member of nicotinamide adenine dinucleotide (NAD)-dependent histone deacetylases. The inhibition of SIRT1 was found to enhance apoptosis in CL1-5 cells treated with *Scutellaria barbata* extract [153]. One of the outcomes of p53 activation is cell cycle arrest, and these mechanisms have potent antitumor effects.

#### 5.2.4. The Activity of Plant Extracts from the Lamiaceae Family as Modulators of PI3K/AKT Signaling

Apoptosis and cell cycle progression are mediated by phosphatidylinositol 3-kinase (PI3K) signal transduction. Activated PI3K is responsible for the conversion of phosphatidylinositol (4,5)-phosphate into phosphatidylinositol (3,4,5)-phosphate [183]. PI3K levels were significantly reduced in A549 cells after the administration of *Nepeta cataria* whole plant extract [184]. That PI3K signaling cascade induces protein kinase B, which is also known as PKB or Akt. Akt is a pro-oncogene and enables tumor proliferation. Akt has numerous downstream effects and controls several biological responses, including the phosphorylation of apoptosis signal proteins Bcl-2 and Bcl-xL followed by the suppression of apoptosis and also caspase-9 and p53. *Prunella vulgaris* root extract was found to modulate the PI3K/Akt signaling pathway in MCF-5 cells [150]. Akt may inactivate mammalian target of rapamycin complex 1 (mTORC1), p70S6 kinase, and p21, which are known to stimulate cell growth and proliferation. The levels of Akt, mTOR, and p70S6K were reduced in A549 cells after treatment with *Rosmarinus officinalis* leaf extract [174]. 

An important suppressor of PI3K is called PTEN: phosphatase and tensin homolog deleted on chromosome 10 [183]. PTEN expression was detected in A549 cells after *Nepeta cataria* whole plant extract [184]. An increased expression of PTEN was also observed in Glc-82 cells after *Salvia miltiorrhiza* root extract treatment, which then resulted in a reduced level of Akt [149]. The levels of phosphorylated Akt were reduced in human prostate cancer PC-3 cells following treatment with *Dracocephalum palmatum* leaf extract [169].

#### 5.2.5. The Activity of Plant Extracts from the Lamiaceae Family as Modulators of NF-κB Signaling

In addition, the stimulation of nuclear factor kappa B (NF-κB) signaling cascade is mainly related to antiapoptotic properties. The NF-κB protein family has five members: p50, p52, p65, RelB, and c-Rel. NF-KB pathways are divided into the canonical and non-canonical. The canonical one is activated by a range of cell stressor molecules such as TNF-α that interact with tumor necrosis factor (TNF) receptors. A first step is the induction of transforming growth factor-β (TGF-β)-activated kinase 1 (TAK1), which promote stimulation of the IkB kinase (IKK) complex composed of IKKγ, IKKα, and IKKβ that phosphorylates IκB, which results in degradation. The remaining NF-κB (p50 and p65) migrate to the nucleus, bind to the DNA, and activate the transcription of numerous genes such as *Bcl-2* and *Bcl-xL*. [185]. Hence, the suppression of NF-κB by *Stachys pilifera* leaf extract is believed to be responsible for the induction of apoptosis in HT-29 cells [154]. The NF-κB signaling cascade also plays a pivotal role in the inflammatory response by activating the transcription of several pro-inflammatory genes such as interleukin *(IL) 1β*, *IL-8, and* cyclooxygenase-2 *(COX-2)* [186]. Commercial standardized *Thymus vulgaris* extract (thymol 0.3% w/w) was found to downregulate the activity of p65 and modulate the release of IL-1β and IL-8, which play roles part in the inflammatory mechanisms in the H460 human lung cancer cell line [187]. COX-2 plays a part in numerous malignancies, including several human cancers. COX-2 is associated with apoptosis suppression followed by uncontrolled proliferation, metastasis, and angiogenesis as a consequence of tumor growth [188]. COX-2 expression was dysregulated by high concentrations of *Origanum majorana* extract in A549 cells [189].

#### 5.2.6. The Activity of Plant Extracts from the Lamiaceae Family as Modulators of Wnt/β-catenin Signaling

CDK/cyclin complexes control Wnt/β-catenin signaling [190]. *Scutellaria barbata* extract is related to decreased the expression of β-catenin in HT-29 cells [179], which is a component of the Wnt/β-catenin signaling pathway responsible for regulating cell growth. The signaling cascade is triggered by binding Wnt ligands to their receptors called Frizzled (Fz) and LDL receptor-related proteins 5 and 6 (LRP5 and LRP6). In the inactive state, a scaffolding protein Axin ensures β-catenin phosphorylation and promotes its degradation, but during the activation of the pathway, the Wnt ligand binds to Fz receptor, and it also allows β-catenin dephosphorylation and accumulation in the nucleus by Axin inhibition and the transcription of Wnt targeted genes such as c-Myc oncogene [191]. The suppression of Wnt signaling cascades induces apoptosis in SW480 and HCT-116 cells [192]. Additionally, *Scutellaria barbata* extract showed a potency to decrease the expression of the c-Myc in human colon adenocarcinoma HT-29 cells; excessive expression results in rapid cellular growth [193]. 

#### 5.2.7. The Activity of Plant Extracts from the Lamiaceae Family as Modulators of Autophagy Signaling

Another type of cell death connected with self-degradation is autophagy. That process may be caused by stress signals that originate from extracellular, intracellular, and endoplasmic reticulum such as growth factor deprivation, nutrient starvation, oxidative stress, protein aggregation, and pathogen infection. Upon autophagy, the organelles named autophagosomes capsules other organelles or a portion of cytosol and then fused them into lysosome and breakdown by lysosomal hydrolases. Nucleation of the phagophore is initiated by the activation of the Unc-51-like kinase 1 (ULK1) complex that triggered the phosphorylation the class IIIPI3K (PI3KC3) complex 1. This step is blocked by Bcl-2 proteins by direct association with becyclin-1, which is a component of that complex. The activated PI3KC complex mediates the production of phospatydyloinositol-3-posphate (PI3P). The first elongation step involves the enrollment of numerous autophagy-related (Atg) proteins by PI3P. The second phase of the elongation step is the formation of autophagosomal membrane-associated protein light chain 3 (LC3)-II from LC3-I via conjugation with phosphatidyl ethanolamine, which revealed the overexpression ratio of LC3-II/LC3-I in H358 and H2087 cells treated with *Scutellaria baicalensis* root extract [165]. Finally, autophagosomes fuse with lysosome and lysosomal enzymes with proteolytic activity degrade its cargo [194,195]. The autophagy is triggered in HT-29 cells by *Origanum majorana* leaf extract [171] and in CL1-5 cells by *Scutellaria barbata* extract [153].

The negative regulator of autophagy is mTOR. mTOR nucleates two distinct multi-protein complexes, mTORC1 and mTORC2. mTORC1 is composed of mTOR, regulatory-associated protein of mTOR (Raptor), mammalian lethal with Sec13 protein 8 (mLST8), proline-rich AKT substrate 40 kDa (PRAS40), and DEP-domain-containing mTOR-interacting protein (Deptor). mTORC1 positively regulates proliferation and cell growth. One of the most important factors involved in the regulation of mTORC1 activity is the tuberous sclerosis complex (TSC1/2). TSC1/2 functions as an activating protein for Rheb (Ras homolog enriched in brain). In turn, Rheb is able to activate mTORC1 [196]. One of the components of the mTOR pathway, Ras small GTPases—Rags, interact with mTORC1 and promote their translocation to a lysosomal membrane where Rheb resides [197].

#### 5.2.8. The Activity of Plant Extracts from the Lamiaceae Family as Modulators of Necrosis Signaling

Necrosis is referred to as accidental cell death. It is frequently detected following harmful physical and chemical conditions, adverse stimuli, or deleterious mutations. The process is mediated by an imbalance of calcium flux, oxidative stress, and PARP. The physiological role of PARP is participation in DNA repair signaling in response to cell injury [198]. Excessive ROS levels, increased intracellular calcium, and the inhibition of PARP result in damage to cell components, degradation of proteins, and DNA damage. Necrosis is characterized by damage of the cell membrane and the release of its components into the extracellular space, resulting in inflammation and damage to the surrounding tissues [199,200]. This pathway is observed to take place in colon adenocarcinoma HT-29 and SW480 cell lines after *Rosmarinus officinalis* extract treatment. Rosemary extract possesses a strong antiproliferative activity, and its mechanism of action suggests the role of excessive intracellular ROS formation [201].

#### 5.2.9. Plant Extracts from the Lamiaceae family and their Impact on Angiogenesis

The critical moment is tumor-induced angiogenesis. *Melissa officinalis* leaf extract halted neovascularization in breast adenocarcinoma MDA-MB-231 cells [202]. Antiangiogenic effects were also exerted by *Prunella vulgaris* root extract in MCF-5 cells [150]. The formation of new blood vessels from pre-existing ones may be regulated by numerous factors. One of them is epidermal growth factor (EGF) and its receptors (EGFR). It was observed that tumor cells demonstrate abnormally high EGFR activity and enhanced sensitivity to their ligands and the progression of tumor [203]. In turn, EGF was found to target vascular endothelial growth factor (VEGF) and induce their activity, whereas VEGF may modulate the EGFR signaling pathway [204]. VEGF demonstrated an ability to increase the permeability of existing blood vessels. The levels of both EGF and VEGF factors were found to be lowered in PC-3 cells and VEGF in DU-145, after *Salvia triloba* extract incubation [205]. *Nepeta cataria* whole plant extract exhibits a preventive effect against A549 cell invasiveness by reducing the level of VEGF [184], whereas *Melissa officinalis* leaf extract acts against PC-3, MCF-7, and A549 cells spread [166]. VEGF may be activated by human telomerase reverse transcriptase (hTERT), specific oncogene, catalytic subunit of the enzyme telomerase essential for chromosome termini replication. hTERT downregulation was observed in PC-3, MCF-7, and A549 cancer cell lines after *Melissa officinalis* leaf extract usage [166].

Other proangiogenic factors are angiogenin (ANG), IL-8 [206], leptin [207], and RANTES (regulated upon activation, normal T-cell expressed and secreted) [208]. ANG is a protein belonging to the RNase A superfamily, whose activity is related to the formation of blood vessels and tumor growth [209], ENA-78 is a chemokine associated with the activation of granulocytic immune cells and vascularity of the tumors, whereas leptin is an endocrine hormone produced by adipocytes. ENA-78 is strongly related to prostate cancer progression [210]. RANTES is an anti-inflammatory chemokine that recruits inflammatory cells and controls the secretion of growth factors included in the angiogenic process [208]. *Salvia triloba* extract was found to have the opposite effect on angiogenesis in PC-3 and DU-145 cells by reducing the levels of VEGF, ANG, ENA-78, IL-8, leptin, and RANTES [205].

### 5.3. The Anti-Cancer Activity of Plant-Derived Compounds from the Lamiaceae Family

Some of the compounds isolated from selected members of the Lamiaceae family and their cytotoxic effect against cancer cell lines are presented in Table 2. This section discusses the molecular mechanisms of action of these phytochemicals against lung, colon, breast, and prostate cancer cell lines. 

#### 5.3.1. The Anticancer Activity of Phenolics Compounds from the Lamiaceae Family

Rosmarinic acid (α-o-caffeoyl-3,4-dihydroxyphenyllactic acid; CAS 20283-92-5), an ester of caffeic acid, and 3,4-dihydroxyphenyllactic acid is commonly found in species of the Lamiaceae family. Rosmarinic acids and their derivatives possess a range of antioxidant, anti-inflammatory, antitumor, anti-angiogenic, and antimicrobial activities, among others [254]. Rosmarinic acid extracted from *Salvia glabra* demonstrates antitumor activity against breast cancer stem-like cells (BCSCs); this has been attributed to apoptosis induction by suppressing the expression of Bcl-2 and increasing that of Bax [255].

Wogonin (5,7-dihydroxy-8-methoxyflavone; CAS 632-85-9) is a flavone with numerous antitumor, anti-inflammatory, antiviral, and neuroprotective properties [256]. A natural source of wogonin is Scutellariae radix, which is the dried root of *Scutellaria baicalensis*. It has been shown to promote both autophagy and apoptosis processes in SW48 cells via the upregulation of autophagic factors including LC3II and Beclin 1 proteins and apoptotic factors such as caspase-3, caspase-8, caspase-9, and Bax proteins. The exposure to wogonin results in G2/M cell cycle arrest and the inhibition of PI3K/Akt signaling through attenuating PI3K protein expression [257]. Wogonin has been found to inhibit the invasiveness of MDA-MB-231 cells via suppressing the synthesis of two key factors related to new blood vessel formation: IL-8 and matrix metallopeptidase-9 (MMP-9) [258]. MMP-9 is critical for the progression of a pro-angiogenic outcome and the release of VEGF during carcinogenesis [259]. Scutellarein (5,6,7,4’-tetrahydroxyflavone), a flavone that is particularly present in the genus *Scutellaria*, has been found to be effective for the prevention and treatment of *Helicobacter pylori* infection, Alzheimer’s disease, and vascular complications of diabetes; it has also been found to inhibit certain carcinomas [260]. It was found that the exposure of the HCT116 cells to the scutellarein extracted from *Scutellaria barbata* induces apoptosis via ROS-mediated mitochondrial membrane permeability and cytochrome c release, and by downregulating the expression of Bcl-2, increasing Bax and cleaved-caspase-3 activity [261].

Another common flavanone is naringenin (4’,5,7-trihydroxyflavanone; CAS 480-41-1). Naringenin has shown antitumor, antioxidant, anti-inflammatory, antiviral, antibacterial, antiadipogenic, and cardioprotective potential [262]. Abaza et al. studied the effect of naringenin isolated from *Thymus vulgaris* whole plant on HTB26, HTB132, SW1116, and SW837 cells. Its administration was found to increase G1/S and G2/M phases cell cycle arrest and apoptotic cell death through the upregulation of p18, p21, Bak, Bax, activation of caspases 3, 7, 8, and 9, and the downregulation of CDK 4, 6, and Bcl2 in all cancer cell lines. It was also found to decrease cell survival factors including PI3K, Akt, and NFκBp65.

#### 5.3.2. The Anticancer Activity of Terpenoids Compounds from the Lamiaceae Family

Arguably, the most important class of chemicals produced by the Lamiaceae family is the terpenoids; of these, the most numerous subclass of compounds with confirmed anticancer properties is that of the diterpenoids. Many possess pro-apoptotic properties, including 3-acetoxylteuvincenone from *Ajuga ovalifolia* whole plants [263], sahandone and sahandol II from *Salvia chloroleuca* roots [264], acetyl-macrocalin B from *Isodon sylvatica* [265], parvifloron D from *Plectranthu ecklonii* [227], and 7β-acetoxy-20-hydroxy-19,20-epoxyroyleanone from *Salvia corrugate* shoots [266]. 3-acetoxylteuvincenone activates Src homology phosphotyrosine phosphatase 2 (SHP2) and induces extracellular signal-regulated kinase (ERK) 1/2 and Akt pathways in A549 cells [263]. SHP2 controls the activation of Akt by insulin-like growth factor-1 (IGF-1), thus regulating the PI3K/Akt pathway related to the blockage of caspase 3-mediated apoptosis [267]. Moreover, SNP2 has been implicated in the activation of ERK 1/2, a member of mitogen-activated protein kinases (MAPKs) that restored the levels of c-Myc [268]. Sahandone and sahandol II change the ratio of Bax/Bcl-2 apoptotic proteins and activate PARP in the MCF-7, LNCaP, and PC-3 cell lines [264]. Acetyl-macrocalin B induces apoptosis in an ROS-dependent manner in A549 cells and then upregulates the p38 MAPK signaling pathway that mediates caspase-9 release. Additionally, the initiation of G2/M cell cycle delay throughout checkpoint kinase (Chks) Chk 1 and Chk 2 activation and cell division cycle 25C (Cdc25C) phosphatase degradation. Chk 1/2 are expressed during the cell cycle, where they regulate the spread of checkpoint signals and promote G2/M arrest by degrading Cdc25C phosphatase [269]. Cdc25C phosphatase dephosphorylates the cyclin B-CDK1 complex and enables cell entry into mitosis but inactivates it, preventing cell cycle progression [270]. Another diterpenoid, parvifloron D, induces apoptotic morphological changes in MDA-MB-231 cells [227]. 7β-acetoxy-20-hydroxy-19,20-epoxyroyleanone promotes G0/G1 cell cycle delay and ROS activation of ERK1/2 kinase in human breast cancer SKBR-3 and human breast carcinoma BT474 cell lines [266].

An important subgroup of terpenoid compounds is tanshinone diterpenoids, which are produced by *Salvia miltiorrhiza* roots; these are also connected with the induction of apoptosis in cancer cells. Increases in apoptosis were observed in colon cancer cells (human colorectal cancer DLD-1, human colon carcinoma COLO 205, and human colorectal adenocarcinoma Caco-2) exposed to trijuganone C [271], prostate cancer cells (PC3 and LNCaP) treated with tanshinone analog 2-(Glycine [methyl ester]methyl)-naphtho [272], and lung (human non-small cell lung cancer PC9 and A549) and breast cancer (MCF-7) cells administered by tanshinone [273]; these changes were attributed to the upregulation of numerous apoptotic factors including cytochrome c, pro- and antiapoptotic proteins ratio, caspases, PARP, p53, and p38. In addition, MCF-7 cells treated with three tanshinones isolated from the roots of *Perovskia abrotanoides* (cryptotanshinone, tanshinone 2A, and hydroxycryptotanshinone) exhibit high amounts of PARP protein cleavage, which is a hallmark of apoptosis [274].

Another diterpenoid, 11α, 12α-epoxyleukamenin E isolated from whole plants of *Salvia cavaleriei* show anticancer potential against HCT116 and SW480 cell lines [275]; this activity was attributed to suppression of the crucial Wnt signaling pathway, which regulates cell fate and the activation of targeted genes, including c-Myc and survivin. The diterpene clerodermic acid found in aerial parts of four *Salvia* species including *S. spinosa*, *S. santolinifolia*, *S. syriaca,* and *S. nemorosa* was also shown to be effective in suppressing Hypoxia-Inducible Factor (HIF) 1 alpha in A549 cells [276], which correlates with tumor metastasis and angiogenesis. Hypoxia is a common microenvironment in many types of solid tumors, and the HIF-1α pathway is crucial for their survival; therefore, the reduction of its expression may serve as a potential cancer therapy target [277].

It has been reported that phlomisoside F, a diterpene glycoside isolated from *Phlomis younghusbandii* root, promotes G0/G1 cell cycle delay and apoptosis induction, as confirmed by the overexpression of caspase-3, caspase-9, and Bax, and the underexpression of Bcl-2 and COX-2 in A549 cells [278]. 2α, 3α, 23-trihydroxy-13α, 27-cyclours-11-en-28-oic acid, a triterpene acid isolated from the aerial parts of *Glechoma longituba*, is responsible for NCI-H460 cell cycle arrest, the induction of ROS-mediated apoptosis, and the nullification of NF-κB activity [279]. The exposure of human lung cancer cell line SK-LU-1 cells to pogostemin A, a meroterpenoid isolated from the aerial parts of *Pogostemon auricularius*, triggered apoptosis and caspase-3 activation [280].

#### 5.3.3. The Anticancer Activity of Polysaccharides Compounds from the Lamiaceae Family

Biologically active polysaccharides from natural plant sources also act as potent antitumor agents. They are also believed to be nontoxic and more preferred for living organisms [281]. SPS2p, a polysaccharide composed of carbohydrates, uronic acid, and proteins from *Scutellaria barbata* demonstrated pro-apoptotic activity against HT29 cells, probably by suppressing the PI3K/Akt pathway [282]. Whereas SBPW3, a polysaccharide containing rhamnose, arabinose, xylose, mannose, glucose, and galactose isolated from the same species also demonstrated anti-metastatic activity via the suppression of EMT in the same cell line [283].

### 5.4. The Anticancer Activity of Essential Oils from the Lamiaceae Family

The members of the Lamiaceae are sources of essential oils. Their cytotoxic potency against cancer cell lines are presented in Table 3. This section discusses their mechanisms of action against lung, colon, breast, and prostate cancer cell lines.

*Nepeta rtanjensis* essential oils rich in trans,cis-nepetalactone induced programmed cell death against A549 and MDA-MB-231 cells [319], as did *Origanum onites* essential oil, which is rich in carvacrol, against Ht-29 cells [320]. *Ocimum viride* essential oils, which have a high content of γ-terpinene, induced apoptosis as a consequence of DNA damage in HT-29 cells [321], while *Thymus revolutus* essential oil with a high content of γ-terpinene and p-cymene induced apoptosis in A549 cells [322]. *Salvia aurea*, *S. judaica,* and *S. viscosa* essential oils containing caryophyllene oxide as a main constituent induced apoptosis triggered by excessive ROS formation in DU-145 cells [323], as did *Zataria multiflora* essential oils in HCT-116 and SW48 cell lines [324]. 

G2/M cell cycle delay and apoptosis were observed in PC-3 cells after exposure to *Lavandula angustifolia* essential oil, with linalool and linalyl acetate as major components [325]. *Plectranthus amboinicus* essential oil, containing high amounts of carvacrol, dysregulated levels of pro- and antiapoptotic factors and induced caspase-9 and caspase-3 in A549 cells [326]. 

Patchouli alcohol, isolated from the essential oil of *Pogostemon cablin,* induced mitochondria-mediated apoptosis and caspase-9 and caspase-3 production in A549 cells [327]. The oil may also demonstrate its anticancer activity by dysregulating the MAPK pathway. *Stachys viticina* essential oils, with their main components being endo-borneol, eucalyptol, and epizonarene, inhibit mediators of apoptosis suppression COX-2 in COLO 205 cells [328]. The caspase-8 and caspase-9-dependent apoptosis pathways are triggered in HT-29 cells by treatment with *Origanum majorana* essential oil containing *inter alia* terpinen-4-ol, alpha-terpinol, alpha-pinene, camphene, p-cymol, B-caryophyllene, bicyclogermacrene, and neophytadiene [329]. *Origanum majorana* essential oil induces non-apoptotic cell death including autophagy and necrosis in HT-29 cells [329]; the same is observed in human lung cancer cell line Calu-3 treatment with *Lavandula dentata* essential oils rich in 1,8-cineole [330].

## 6. Conclusions

Cancer causes the greatest economic burden of any of the top 15 causes of death worldwide, both for the patient and society in general. Therefore, there is a great need to identify new active molecules with anticancer activities. Medicinal plants and their bioactive compounds are widely used in the treatment of numerous diseases. The species of the Lamiaceae are considered important because of their use in traditional medicine throughout the world. The Lamiaceae include a range of secondary metabolites including polyphenols, terpenoids, alkaloids, or essential oils that demonstrate promising cytotoxic activity against lung, breast, prostate, and colorectal cancer cell lines mainly via the apoptosis pathway by the modulation of cell cycle progression, changes in genes expression, and impact on various signaling cascades. These biologically active compounds represent promising candidates for supportive use in anticancer therapy; however, further extensive scientific and clinical investigations are required. 

## Figures and Tables

**Table 1:** Cytotoxic properties of selected extracts from the Lamiaceae family against lung, colon, breast, and prostate cancer cells.

Name of The Species	Part of the Plant	Type of Extract	Class of Compounds/Compounds Identified in Extract	Cancer Cell Lines	Ref.
*Ajuga chamaepitys* subsp. chia (Schreb.) = synonym of *Ajuga chia* (Schreb)	aerial parts	ethanolic	-	T-47D	[103]
*Cyclotrichium niveum* (Boiss.) Manden. and Scheng	aerial parts	ethyl acetate	gallic acid, protocatechuic acid, chlorogenic acid, caffeic acid, gentisic acid, p-coumaric acid, ferulic acid, rutin, luteolin-7 glucoside, quercetin, luteolin, apigenin	MCF-7	[104]
*Eremostachys azerbaijanica* Rech.f.	rhizomes	methanolic, n-hexane, dichloromethane	fatty acid derivatives and steroids steroids and derivatives, heterocyclic hydrocarbons, sesquiterpenes, and linear alkanes	A549	[105]
*Eremostachys azerbaijanica* Rech.f.	rhizomes	dichloromethane	steroids and derivatives, heterocyclic hydrocarbons, sesquiterpenes, and linear alkanes	HT-29	[105]
*Hymenocrater platystegius* Rech.f.	leaves and flowers	aqueous	phenolics	MCF-7	[106]
*Hyptis pectinata* L.	leaves	ethanolic	*-*	MCF-7	[107]
*Hyptis pectinate* L.	leaves, branches, root	ethanolic	pectinolide J, hyptolide, and pectinolide E	MDA-MB-231	[108]
*Lavandula coronopifolia* Poir.	aerial parts	petroleum ether, ethyl acetate, chloroform, and ethanol	-	MDA-MB-231	[109]
*Lavender angustifolia* Mill	leaves	methanolic	phenolics, flavonoids, glycosides	MCF-7	[110]
*Melissa officinalis* L.	leaves	ethanolic	phenolics and flavonoids	MCF-7, PC-3 and A549	[111]
*Melissa officinalis* L.	leaves	ethanolic	phenolics: 3-(3,4-dihydroxyphenyl)-lactic acid, caftaric acid, caffeic acid hexoside, fertaric acid, caffeic acid, sulphated rosmarinic acid, yunnaneic acid E, lithospermic acid A isomer, chicoric acid, yunnaneic acid F, salvianolic acid C derivative I, salvianolic acid C derivative II, rosmarinic acid hexoside, sagerinic acid, rosmarinic acid, salvianolic acid A, salvianolic acid C derivative III, lithospermic acid A, salvianolic acid A isomer, salvianolic acid C derivative IV	NCI-H460	[112]
*Nepeta bracteata* Benth.	flowering shoot	aqueous	phenolics	MCF-7	[106]
*Ocimum* *americanum* L.	leaves	ethyl acetate	phenolics, flavonoids, flavanols, tannins, saponins	HCT-116	[113]
*Ocimum basilicum var. thyrsiflorum* (L.) Benth.	leaves	ethanolic	phenolics and flavonoids: cinnamic acid, gallic acid, methylgallate, ellagic acid, methyl ellagic acid, apigenin, luteolin, vitexin, isovitexin	HCT-116	[114]
*Origanum dayi* Post.	aerial parts	ethanolic	-	MCF-7 and T47D	[103]
*Origanum majorana* L.	aerial parts	ethyl acetate	2-(4-hydroxy phenyl) ethanol, vanillic acid, 4-hydroxybenzoic acid, syringic acid, caffeic acid, vanillin, trans-ferulic acid, luteolin, cinnamic acid	MDA-MB-231 and HT-29	[115]
*Origanum syriacum* L.	leaves	ethanolic	-	LoVo and SW620	[116]
*Orthosiphon aristatus* (Blume) Miq.	leaves	methanolic	phenolics	MCF-7	[117]
*Orthosiphon pallidus* Royle ex Benth.	whole plants	aqueous	-	MCF-7 and MDA-MB-231	[118]
*Phlomis viscosa* Poiret.	leaves, flowers and stems	ethanolic	diosmin, isovaleraldehyde, 2,4-hexadienal, 2-hexenal, alpha terpinene, 1-octen-3-ol, himachala-2,4-diene, n-octanal, buorbonene, 1-propanal, 2-methyl, cubebene	MCF-7	[119]
*Plectranthus amboinicus* (Lour.) Spreng	leaves	chloroform	diterpene/7-acetoxy-6-hydroxyroyleanone	MCF-7	[120]
*Plectranthus amboinicus* (Lour.) Spreng.	leaves	ethanolic	-	MCF-7	[121]
*Pogostemon heyneanus* Benth.	leaves	chloroform	-	MCF-7 and MDA-MB-231	[121]
*Rosmarinus officinalis* L.	leaves	ethanolic	flavonoids, diterpenes, triterpenes: apigenin, hispidulin, cirsiliol, diosmetin, cirsimaritin, rosmanol, epiisorosmanol, epirosmanol, genkwanin, miltipolone, carnosol, rosmadial, anemosapogenin, rosmaridiphenol, augustic acid, benthamic acid, carnosic acid, 12-methoxycarnosic acid, shogaol, micromeric acid, hinokione, betulinic acid, ursolic acid	HT-29 and SW480	[122]
*Rosmarinus officinalis* L.	leaves and flowers	aqueous	phenolics	MCF-7	[106]
*Rosmarinus officinalis* L.	aerial parts	aqueous	*-*	Caco-2	[123]
*Rosmarinus officinalis* L.	leaves	methanolic	terpenes	LoVo	[124]
*Rosmarinus officinalis* L.	leaves	methanolic	phenolics and flavonoids, alkaloids, tannins, glycosides	MCF-7	[110]
*Rosmarinus officinalis* L.	leaves	ethanolic	phenolics: protocatechuic acid, caffeic acid, ellagic acid, ferulic acid, rosemarinic acid, carnosol, carnosic acid	MCF-7	[125]
*Salvia* *fruticosa* Mill subsp. *thomasii*	aerial parts	methanolic	luteolin, luteolin 7-O-glucoside, rutin, salvigenin	MCF-7, MDA-MB-231, RKO and Caco-2	[126]
*Salvia ballotiflora* Benth.	aerial parts	chloroform	-	A549	[127]
*Salvia fruticosa* Mill	bark	methanolic	-	MCF-7, T-47D and MDA-468	[128]
*Salvia fruticosa* Mill. (SF.)	aerial parts	aqueous	phenolics and flavonoids: rosmarinic and caffeic acid	Caco-2 and HT-29	[129]
*Salvia hispanica* L.	seeds	ethanolic	-	A549	[130]
*Salvia hispanica* L.	seeds	ethanolic	tannins, saponins, flavonoids, alkaloids, proteins, phenols	PC-3	[131]
*Salvia officinalis* L.	leaves	ethanolic	eucalyptol (1,8-cineole), α-thujone, β-thujone, camphor, β-caryophyllene, α-caryophyllene (α-humulene), viridiflorol, manool	A-549, HT-29	[132]
*Salvia officinalis* L.	leaves	aqueous	phenolics: caffeic acid, syringic acid, rutin, coumaric acid, vanillin, quercetin, cinnamic acid	MDA-MB-231	[133]
*Salvia pilifera* Montbret and Aucher ex Benth.	whole plant	methanolic	fumaric acid, gallic acid, gallocatechin, catechin, oleorufein, 4-hydroxybenzoic acid, caffeic acid, syringic acid, ellagic acid, 3-hydroxy cinnamic acid and protocatechuic acid	DU-145	[134]
*Salvia pilifera* Montbret et Aucher ex Benth.	mericarps	ethanolic	-	A549	[135]
*Salvia verbenaca* L.	leaves	ethyl acetate	flavonoids and terpenes	MDA MB-231	[136]
*Satureja horvatii* subsp. *macrophylla* (Halácsy) Baden.	aerial parts	methanolic	monoterpene hydrocarbons, oxygenated monoterpens, sesquiterpene hydrocarbons, oxygenated sesquiterpenes, monoterpenes, sesquiterpenes: α-thujene, α-pinene, camphene, 1-octen-3-ol, α-myrcene, 3-octanol, α-phellandrene + δ 3-carene, α-terpinene, p-cymene, limonene, 1,8-cineole, cis-ocimene, γ-terpinene, trans sabinene hydrate, terpinolene, linalool, trans-pinocarveol, cis-verbenol, camphor, borneol, terpinene-4-ol, p-cymen-8-ol, α-terpineol, dihydrocarvone, thymol methyl ether, thymoquinone, thymol, carvacrol, caryophyllene, aromadendrene, α-humulene, alloaromadendrene, γ-muurolene, viridiflorene, γ-elemene, b-bisabolene, γ-cadinene, δ-cadinene, (-)-spathulenol, caryophyllene oxide, viridiflorol	A549	[137]
*Sideritis ozturkii* Aytaç and Aksoy	flower and leaf	methanolic	gallic acid, protocatechuic acid, catechin, 4-hydroxybenzoic acid, caffeic acid, syringic acid, rutin trihydrate, trans-p-coumaric acid, trans-ferulic acid, myricetin, trans-resveratrol, quercetin, trans-cinnamic acid, naringenin, kaempferol	DLD-11	[138]
*Sideritis syriaca* L.	leaves	methanolic	phenolic acids: gallic acid, p-hydroxybenzoic acid, cafeic acid, chlorogenic acid, p-coumaric acid, ferulic acid, o-coumaric acid, rosmarinic acid and trans- cinnamic acid	MCF-7	[139]
*Teucrium fruticans* L.	leaves	ethanol/ethyl acetate/water	-	A549	[140]
*Thyme vulgaris* L.	leaves	methanolic	phenolics and flavonoids, tannins	MCF-7	[110]
*Thymus daenensis* Celak.	leaves and stems	ethanolic	-	MCF-7	[141]
*Thymus mastichina* L.	whole plant	ethanol/ethyl acetate/water	-	A549	[140]
*Vitex trifolia* L.	leaves	methanol	-	MCF-7	[142]

**Table 2:** Cytotoxic properties of isolated pure compounds from the Lamiaceae family against lung, colon, breast, and prostate cancer cells.

Name of The Species	Part of the Plant	Active Compounds/ Class of Compounds	Cancer Cell Lines	Ref.
*Caryopteris incana* (Thunb.) Miq.,	whole plant	abietane diterpenes: caryopincaolide A, C, D	A549, Calu-1	[211]
*Clerodendranthus spicatus* (Thunb.) C. Y. Wu	aerial parts	ursane-type triterpenoids: spicatusoids A–E and three known ones and a known oleanane-type triterpenoid	A-549, MCF-7, SW480	[212]
*Clerodendrum indicum* (L.) Kuntze and *Clerodendrum villosum* Blume	roots	3b-hydroxy-D:B-friedo-olean-5-ene, oleanolic acid 3-acetate, taraxerol, lupeol, betulinic acid, (22E)-stigmasta-4,22,25-trien-3-one, stigmasta-4,25-dien-3-one, stigmasta-4,22-dien-3-one, 22-dehydroclerosterol, clerosterol, stigmasterol, b-sitosterol, pectolinarigenin, hispidulin	SW620, ChaGo-K-1, BT474	[213]
*Clerodendrum inerme* (L.) Gaertn	leaves	harwickiic acid, Crolerodendrum B-abietane diterpenes, uncinatone, acacetin, kaempferol 3,7,4′-trimethyl ether, 14,15-dihydro-15β-methoxy-3-epi-caryoptin, 5α,8α-epidioxyergosta-6,22-diene-3β-ol	HCT-116	[214]
*Clerodendrum kiangsiense* Merrill ex H. L. Li	herb	abeo-abietane diterpenoid: 12-methoxy-6,11,14,16-tetrahydroxy-17(15Ñ16)-abeo5,8,11,13-abietatetraen-3,7-dione, cryptojaponol, fortunin E	A549, MCF-7	[215]
*Clerodendrum yaundense* Gurke	twigs	lupane-type triterpene: (16a)-lupa-12,20(29)-dien-16-ol (clerodendrumol) and, O-Acetylclerodendrumol (16a)-Lupa-12,20(29)-dien-16-yl Ace-tate	MDA-MB-231	[216]
*Isodon excisoides* (Sun ex C. H. Hu) H. Hara, J. Jap	aerial parts	1α,7α,14β-trihydroxy-20-acetoxy-ent-kaur-15-one, henryin, reniformin C; kamebacetal A	HCT-116, NCI-H1650	[217]
*Isodon rubescens* (Hemsl.) H. Hara	aerial parts	ent-7,20-epoxy-kaur-16-en-1α,6β,7β,15β-tetrahydroxyl-11-O-β-d-glucopyranoside, ent-7,20-epoxy-kaur-16-en-6β,7β,14β,15β-tetrahydroxyl-1-O-β-d-glucopyranoside, ent-7,20-epoxy-kaur-16-en-6β,7β,15β-trihydroxyl-1-O-β-d-glucopyranoside, andent-7,20-epoxy-kaur-16-en-7β,11β,14α,15β-tetrahydr-oxyl-6-O-β-d-glucopyranoside, sodonterpene II, enmenol-1-β-glucoside, andenmenol	HCT-116	[218]
*Isodon phyllostachys* (Diels) Kudo	aerial parts	enmein-type diterpenoids enmein-type diterpenoids: 20-episerrin C, serrin C, isodocarpin, serrin B	A549, MCF-7, SW480	[219]
*Isodon rubescens* (Hemsl.) H.Hara	leaves	diterpenoids: 6-epi-11-O-acetylangustifolin and 11-O-acetylangustifolin	A549	[220]
*Isodon wikstroemioides* (Hand.–Mazz.) H. Hara	aerial parts	diterpenoids: 11, 20-epoxy-ent- kauranoids, isowikstroemins H–M, along with two known analogues, macrocalyxin B	A549, MCF-7	[221]
*Leonurus japonicus* Houtt.	fruits	leonuronins A and B	A549	[222]
*Ocimum basilicum* var.thyrsiflorum (L.) Benth	leaves	C-glycosylated derivative of apigenin: 3′′-O-acetylvitexin	HCT-116	[114]
*Ocimum sanctum* L.	aerial parts	lignans: (-)-rabdosiin	MCF-7, SKBR3, HCT-116	[201]
*Orthosiphon stamineus* Benth.	aerial parts	7,4′-dimethylkaempferol, 5,7,4′-trimethylkaempferol, 7,3′,4′-trimethylquercetin, 5,7,3′,4′-tetramethylquercetin, 5,7,4′-trimethylquercetin, 3,5,3′,4′-tetramethylquercetin, 5,7,3′,6′-tetramethoxyflavone, 5,7,3′,4′-tetramethoxyflavone, 2*S*-5,6,7,3′,4′-pentamethoxyflavanone, 2*S*-5′-hydroxy-5,7,3′,4′-tetramethoxyflavanone	MCF-7, MDA-MB-231	[223]
*Phlomis bruguieri* Desf	aerial parts	4’-methoxy-luteolin-7-phosphate	MCF-7	[224]
*Phlomoides umbrosa* (Turcz.) Kamelin & Makhm	roots	28-noroleanane-derived spirocyclic triterpenoids: phlomisu D, phlomisu E, (2α,3α,17R,18β)-19(18→17)-abeo-28-norolean-12-ene-2,3,18,23,24-pentol, phlomispentaol	MCF-7	[225]
*Plectranthus cylindraceus* Hoechst. Ex. Benth	aerial parts	sesquiterpene: plectranol A, maaliol, penduletin and chrysosplenol D	MBD-MB-321	[226]
*Plectranthus ecklonii* Benth.	whole plant	abietane diterpenoid: parvifloron D	MDA-MB-231	[227]
*Plectranthus madagascariensis* Benth	whole plant	royleanone diterpenes: 7α-formyloxy-6β-hydroxyroyleanon, 7α,6β-dihydroxyroyleanon, 7α-acetoxy-6β-hydroxyroy-leanone	MDA-MB-231, MCF-7, HCT-116, NCI-H460	[228]
*Plectranthus scutellarioides* (L.) R. Br.	leaves	diterpenoids: spiroscutelones A–C	MCF-7	[229]
*Pogostemon auricularius* L. Hassk.	aerial parts	meroterpenoids with pyrone-sesquiterpenoid hybrid skeletons: pogostemins A-C	SW480, SK-LU-1	[230]
*Pogostemon cablin* (Blanco) Benth	aerial parts	phenylethanoid glycosides: verbascoside, pedicularioside G ()	A549, HCT-15	[231]
*Premna odorata* Blanco	bark	tetrahydrofurofuran lignin: 4β-hydroxyasarinin	HT-29, MCF-7	[232]
*Rabdosia serra* (Maxim.) Hara	grasses	diterpenoids: rabdosins E–K, serrin B, serrin A, isodocarpin and lushanrubescensin J	A549, NCI--H661	[233]
*Salvia ballotiflora* Benth	leaves	diterpenoids: 7α-acetoxy-6,7-dihydroicetexone, anastomosine	SK-LU-1	[234]
*Salvia ballotiflora* Benth.	aerial parts	diterpenes: 19-deoxyicetexone,7,20-dihydroanastomosine, icetexoneand19-deoxyisoicetexone,	A549, MCF-7	[127]
*Salvia lachnocalyx* Hedge	shoots	(2*Z*,6*Z*,10*Z*,14*E*)-geranylfarnesol and spathulenol	MCF-7, HT-29	[235]
*Salvia leriifolia* Benth.	aerial parts	labdane diterpenoids: 6β,13β-dihydroxylabd-8, 14-diene-19-oic acid, 13-hydroxylabd-8, 14-diene-6β,19-olide, 8,12E,14-labdatrien-6,19-olid	MDA-MB-231, DU 145	[236]
*Salvia leucantha* Cav	aerial parts	neoclerodane diterpenoids: leucansalvialins FeI (1–4), and one rare 18(4→3)-abeo-abietane diterpenoid, leucansalvialin J	A549, MCF-7, SW480	[237]
*Salvia officinalis* L.	leaves	diterpene: manool	MCF-7	[238]
*Salvia sahendica* Boiss. & Buhs	leaves	terpenoid: sclareol	MDA-MB-231	[239]
*Salvia santolinifolia* Boiss	callus	salvialactomine, 5-Methylflavone	PC-3	[240]
*Salvia tebesana* Bunge	roots	diterpenoids: tebesinone A (1) and tebesinone B (2), aegyptinone A (3) andaegyptinone B (4)	MCF-7, PC-	[241]
*Salvia tiliifolia* L.	aerial parts	neo-clerodane diterpenoids: tiliifolins A–E	A549, MCF-7, SW480	[242]
*Salvia urmiensis* Bunge	aerial parts	triterpenoids: urmiensolide B and urmiensic acid	MCF-7	[243]
*Scutellaria barbata* D. Don	whole plant	neoclerodane diterpenoids: barbatin F, barbatin G, scutebata A, scutebata B, scutebata C and scutebata P	LoVo, MCF-7, HCT-116	[244]
*Scutellaria barbata* D. Don	aerial parts	neo-clerodane diterpenoids: 13(R*)-1β,6α-dibenzoyloxy-7β-hydroxy-8β,13-epoxy-3-neo-cleroden-15,16-olide (scutebata C1), 2-oxo-6α-nicotinoyloxy-7β-benzoyloxy-8β-hy-droxy-3,11(E),13-neo-clerodatrien-15,16-olide.(scutebata X), 13(R*)-2-oxo-6α-acetoxy-7β-nicotinoyloxy-8β,13-epoxy-3-neo-cleroden-15,16-olide, (scutebata A1)	MCF-7, A549	[245]
*Scutellaria barbata* D. Don	aerial parts	neoclerodane diterpenoid: barbatin H, scutebata P, scutebarbatine F, 6-O-nicotinoylscutebarbatine G, scutebata G, scutebata E, scutebata D, barbatin C, scutebarbatine A, scutebartine G, scutebarbatine B, 6,7-Di-O-acetoxybarbatin A, scutebata C, scutebata A, scutebarbatine X, scutebata B	LoVo, MCF-7, HCT-116	[246]
*Scutellaria barbata* D.Don	whole plant	neo-clerodane diterpenoids: scutebarbatolides A-C, 4-deoxy-11,12-didehydroandrographolide, scutehenanine H, 14β-hydroxyscutolideK	LNCaP, MCF-7	[247]
*Scutellaria coleifolia* Levl.	aerial parts	neo-clerodane type diterpenoids: scutefolides G1-S	A549, MCF-7	[248]
*Scutellaria strigillosa* Hemsley, J. Linn. Soc	whole plant	neo-clerodane diterpenoids: scutestrigillosins A-C	MCF-7, HT-29	[249]
*Teucrium polium* L.	aerial parts	saponin glycosides: poliusaposide A, poliusaposide B, poliusaposide C	MDA-MB-468, HCC-2998, COLO 205	[250]
*Teucrium ramosissimum* Desf.	leaves	sesquiterpene: β-eudesmol	A549, HT-29, Caco-2	[251]
*Teucrium viscidum* Blum.	whole plants	abietane diterpenoid: teuvisone, a pair of new dimeric abietane diterpenoid stereoisomers: biteuvisones A and B, and three sesquiterpenoid lactones, teuvislactones A−C	teuvisone and biteuvisones B showed cytotoxic effect against NCI-H460, HCT-116	[252]
*Thymus alternans* Kloko.	aerial parts	triterpenes: 3a-hydroxy-urs-12,15-diene, a-amyrin, b-amyrin, isoursenol, epitaraxerol, and oleanolic acid	MDA-MB-231, HCT-15 HCT-116, U1810	[253]

**Table 3:** Cytotoxic properties of selected essential oils from the Lamiaceae family against lung, colon, breast, and prostate cancer cells.

Name of The Species	Part of the Plant	Compounds Identified in Essential Oils	Cancer Cell Lines	Ref.
*Anisomeles indica* Kuntze.	leaves	The major compounds: farnesyl acetone (10.67%), nootkatone (8.35%), phytol acetate (7.35%), jasmatone (7.81%)	A549	[284]
*Ballota undulata, Ballota saxatilis Ballota nigra* ssp. foetida	aerial parts	*Ballota nigra* ssp. foetida: germacrene D (23.1%), (E)-β-caryophyllene (20.3%) and caryophyllene oxide (6.2%), *Ballota saxatilis*: linalool (11.2%), (E)-β-caryophyllene (8.8%), caryophyllene oxide (6.3%) and (E)-2-hexenal (5.6%), *Ballota undulata*: germacrene D (16.0%), and bicyclogermacrene (10.4%)	MCF-7	[285]
*Cantinoa stricta* (Benth.) Harley and J.F.B. Pastore (*formely Hyptis stricta* Benth.)	leaves and flowers	The major compounds: caryophyllene oxide (leaf –31.6%; flower –21.7%) and cis-pinane (leaf –15.4%; flower –9.7%), α-pinene (9.4%) and β-pinene (9.1%)	MCF-7, NCI-H460, PC-3	[286]
*Cedronella canariensis* *var. canariensis* (L.) Webb and Berthel.	aerial parts	The major compounds: pinocarvone (58.0%) and β-pinene (10.8%)	MDA-MB-231, HCT-116	[287]
*Cunila angustifolia* Benth.	leaves	The major compounds: pulegone (29.5%), isomenthol (27.0%), menthone (8.6%), neomenthol (7.2%), menthyl acetate (2.5%), and caryophyllene oxide (2.0%)	A-549, MCF-7	[288]
*Elsholtzia ciliata* (Thunb.) Hylander	aerial parts	The major compounds: dehydroelsholtzia ketone, elsholtzia ketone, sesquiterpenes β-bourbonene, caryophyllene, α-caryophyllene, germacrene D, and α-farnesene	MDA-MB-231	[289]
*Lavandula hybrid* Rev., *Lavandula latifolia* Medikus., *Lavandula* vera D.C. *and Lavandula angustifolia* Miller.	aerial parts	Terpenes: linalool and linalyl acetate terpenoids: 1,8-cineole	Caco-2	[290]
*Leonotis nepetifolia* (L.) R.Br.	leaves	The major compounds: 3-octanone (3.75%), (E)-ocimene (15.85%), (Z)-ocimene (7.01%), linalool, caryophillene oxide, and 1-octen-3-ol (42.58%)	HCT-116	[291]
*Melissa officinalis* L.	leaves	citral (47.2%), caryophyllene oxide (10.2%), citronellal (5.4%), geraniol (6.6%), geranyl acetate (4.1%) and β- caryophyllene (3,8%)	MCF-7, NCI-H460	[292]
*Mentha citrate* Ehrh.	aerial part	The major compounds: linalool (34.69%), linalyl acetate (35.75%)	HCT-116	[293]
*Mentha spicata* L.	aerial parts	The major compounds: carvone (49.5%), limonene (16.1%), 1,8-cin-eole (8.7%), cis-dihydrocarvone (3.9%), β-caryophyllene (2.7%), germacrene D (2.1%), and β-pinene (1.1%)	HCT-116, MCF-7	[294]
*Meriandra dianthera* (Konig ex Roxb.) Benth.	aerial parts	camphor (54.3%), 1,8-cineole (12.2%), and camphene (10.4%)	MCF-7, LoVo	[295]
*Nepeta menthoides* Boiss. and Bohse.	-	The major compounds: 1,8-cineole (70.06%)	MCF-7, T-47D, MDA-MB-231	[296]
*Nepeta schiraziana* Boiss.	aerial parts	The major compounds: 1,8-cineole (33.67%), germacrene D (11.45%), β-caryophyllene (9.88%), and caryophyllene oxide (7.34%)	MCF-7	[297]
*Nepeta sintenisii* Bornm.	aerial parts	4aα,7α7aβ nepetalactone (51.74%), β-farnesene (12.26%), 4aα,7α,7aα nepetalactone (8.01%), germacrene-D (5.01%), and 4aα7β,7aα-nepetalactone (3.71%)	LS180, MCF-7	[298]
*Ocimum basilicum L.*, *Mentha spicata* L.	chopped leaves and stems	*Mentha spicata*: carvone (85.4%), limonene (8.4%), and β-Pinene (1.4%) *Ocimum basilicum*: methyl chavicol (74.9%), linalol (18.4%)	MCF-7, Caco2	[299]
*Origanum majorana* L.	aerial parts	The major compounds: terpinen-4-ol (23.2%), Cis-sabinene hydrate (17.5%), γ-terpinene (10.5%), p-cymene (9%), α-terpineol (5.6%), α-terpinene (4.7%), and trans-sabinene hydrate (4.0%)	HT-29	[300]
*Origanum dictamnus* (L.) Kostel., *Origanum libanoticum* Boiss., and *Origanum microphyllum* (Bentham) T. Vogel	-	The major compounds: carvacrol, p-cymene, linalool, γ-terpinene, and terpinen-4-ol as	LoVo	[301]
*Origanum vulgare* L.	leaves	pulegone (77.4%), menthone (4.8%), cis-Isopulegone (2.2%), piperitenone (2.1%), limonene (1.0%), and myrcene (0.6%)	MCF-7, HT-29	[302]
*Plectranthus. cylindraceus* Hocst. ex Benth., *Plectranthus. asirensis* JRI Wood. and *Plectranthus barbatus* Andrews.	leaves and branches	The major compounds: α-pinene (46.2%), maaliol (42.8%), and β-caryophyllene (13.3%)	HT-29	[303]
*Premna microphylla* Turcz.	aerial parts	The major compounds: blumenol C (49.7%), β-cedrene (6.1%), limonene (3.8%), α-guaiene (3.3%), cryptone (3.1%), and gurkeα-cyperone (2.7%)	MCF-7	[304]
*Rosmarinus officinalis* L.	aerial parts	The major compounds: 1,8-cineole (23.56%), camphene (12.78%), camphor (12.55%), and β-pinene (12.3%)	MCF-7	[305]
*Salvia macrosiphon* Boiss.	aerial parts	The major compounds: linalool (19%), β-cedrene (14.64%), and β-elemene (13.33%)	MCF-7, MDA-MB-231, T-47D	[306]
*Salvia officinalis* L.	aerial parts	The major compounds: α-thujone, 1,8-cineole, and camphor	LNCaP, MCF-7	[307]
*Salvia officinalis* L.	aerial parts	α-thujone (29.39%), 1,8-cineole (eucalyptol 22.8%), and camphor (13.05%)	Caco-2, HT-29, HCT-116	[308]
*Salvia ringens* Sibth. and Sm.	whole plant	The major compounds: 1.8-cineole (31.99%), cam-phene (17.06%), borneol (11.94%), and α-pinene (11.52%)	HCT-116	[309]
*Satureja intermedia* C.A.Mey	aerial parts	γ-terpinene (37.1%), thymol (30.2%), p-cymene (16.2%), limonene (3.9%), α-terpinene (3.3), myrcene (2.5%), germacrene B (1.4%), elemicine (1.1%), and carvacrol (0.5%)	MCF-7	[310]
*Satureja thymbra* L. *and Satureja parnassica* Heldr. and Sart. ex Boiss	aerial parts	The major compounds: carvacrol, thymol, γ-terpinene, and p-cymene	MCF-7, A549	[311]
*Stachys annua* L. subsp. annua	aerial parts	The major compounds: phytol (9.8%), germacrene D (9.2%), and spathulenol (8.5%)	HCT-116, MDA-MB-231	[312]
*Stachys annua* L. subsp. *annua*	aerial parts	phytol (9.8%), germacrene D (9.2%), and spathulenol (8.5%)	MDA-MB 231, HCT-116	[312]
*Stachys parviflora* L.	aerial parts	The major compounds: α-terpenylacetate (23.6%), caryophyllene (16.8%), bicyclogermacrene (9.3%), spathulenol (4.9%), and α-pinene (4.2%)	HCT-116	[313]
*Tetradenia riparia* (Hochst.) Codd.	leaves	fenchone (6.1%), dronabinol (11.0%), aromadendrene oxide (14.7%), and (E,E)–farnesol (15.0%)	HT-29, MCF-7	[314]
*Thymus alternans* Klokov.	aerial parts	The major compounds: (E)-nerolidol, neryl acetate, nerol	MDA-MB-231, HCT-15, HCT-116, U1810	[253]
*Thymus munbyanus* subsp. Coloratus (Boiss. and Reut.) Greuter and Burdet	stems, leaves	borneol (44.8 and 31.2%) Other components occurring in noteworthy levels were camphor (5.7 and 13.6%), camphene (3.6 and 7.5%), 1,8-cineole (6.0 and 4.2%), and germacrene D (5.0 and 3.1%)	MDA-MB-231	[315]
*Zataria multiflora* Boiss.	aerial parts	-	MCF-7	[316]
*Zataria multiflora* Boiss. *Satureja bakhtiarica* Bunge.	leaves	The major compounds: phenol (56.35%, 37.4%), thymol (13.82%, 22.6%), P-cymene (8.79%, 19.3%), γ-terpinene (3.36%, 5.0%), β-myrcene (1.91%, 0.8%), β-caryophyllene (1.28%, 2.2%), α-terpinene (1.21%, 0.9%), caryophyllene oxide (0.47%, 2.0%), and carvacrole (2.88%, 0.2%)	MDA-MB-231	[317]
*Zhumeria majdae* Rech.f. and Wendelbo.	aerial parts	The major compounds: linalool (24.4–34.6%), camphor (26.1–34.7%), and translinalool oxide (7.6–28.6%)	MCF-7	[318]

## Abbreviations

Akt	Protein Kinase B
ANG	Angiogenin
Apaf	Apoptotic Protease Activating Factor
Atg	Autophagy-Related Protein
Cdc25C	Cell Division Cycle 25C Phosphatase
CDKs	Cyclin-Dependent Kinases
COX	Cyclooxygenase
Deptor	DEP-Domain-Containing mTOR-Interacting Protein
DISC	Death-Inducing Signaling Complex
EGF	Epidermal Growth Factor
EGFR	Epidermal Growth Factor Receptor
ERK	Extracellular Signal-Regulated Kinase
FADD	Fas-Associated Death Domain
Fz receptor	Frizzled Receptor
HIF	Hypoxia-Inducible Factor
hTERT	Human Telomerase Reverse Transcriptase
IKK	IkB Kinase
IL	Interleukin
LC3	Autophagosomal Membrane-Associated Protein Light Chain 3
MAPK	Mitogen-Activated Protein Kinase
mLST8	Mammalian Lethal with Sec13 Protein 8
MMP	Matrix Metallopeptidase
mTOR	Mammalian Target of Rapamycin
mTORC1	Mammalian Target of Rapamycin Complex 1
NF-κB	Nuclear Factor Kappa B
PARP	Poly(ADP-ribose) Polymerase
PI3K	Phosphatidylinositol 3-Kinase
PI3KC3	Class IIIPI3K Complex 1
PI3P	Phospatydyloinositol-3-Posphate
PRAS40	Proline-Rich AKT Substrate 40 kDa
PTEN	Phosphatase and Tensin Homolog
RANTES	Regulated Upon Activation, Normal T-cell Expressed and Secreted
Raptor	Regulatory-Associated Protein of mTOR
Rheb	Ras Homolog Enriched in Brain
ROS	Reactive Oxygen Species
SHP2	Src Homology Phosphotyrosine Phosphatase 2
SIRT1	Sirtuin
TAK	TGF-β-Activated Kinase
TNF	Tumor Necrosis Factor
TRAIL	TNF-Related Apoptosis-Inducing Ligand
ULK1	Unc-51-like Kinase 1 Complex
VEGF	Vascular Endothelial Growth Factor
